# Endolysosomal Cation Channels and MITF in Melanocytes and Melanoma

**DOI:** 10.3390/biom11071021

**Published:** 2021-07-13

**Authors:** Carla Abrahamian, Christian Grimm

**Affiliations:** Walther Straub Institute of Pharmacology and Toxicology, Faculty of Medicine, Ludwig-Maximilians-University, 80336 Munich, Germany; carla.abrahamian@lrz.uni-muenchen.de

**Keywords:** TPC, two-pore, lysosome, TPC1, TPC2, TRPML, mucolipin, MCOLN, TRPML1, MITF, melanocytes, melanoma, mTOR, TFEB, calcium

## Abstract

Microphthalmia-associated transcription factor (MITF) is the principal transcription factor regulating pivotal processes in melanoma cell development, growth, survival, proliferation, differentiation and invasion. In recent years, convincing evidence has been provided attesting key roles of endolysosomal cation channels, specifically TPCs and TRPMLs, in cancer, including breast cancer, glioblastoma, bladder cancer, hepatocellular carcinoma and melanoma. In this review, we provide a gene expression profile of these channels in different types of cancers and decipher their roles, in particular the roles of two-pore channel 2 (TPC2) and TRPML1 in melanocytes and melanoma. We specifically discuss the signaling cascades regulating MITF and the relationship between endolysosomal cation channels, MAPK, canonical Wnt/GSK3 pathways and MITF.

## 1. Introduction

Melanocytes are neural-crest derived cells that produce melanin, the primary determinant of skin color. Melanin is also found in hair, in the iris of the eye, and in the stria vascularis of the inner ear and, to a lesser degree, in a broad range of other tissues throughout the body [1]. There are two major types of melanin called eumelanin (dark, brown, black) and pheomelanin (yellow, red, light brown). Melanin is produced and stored in melanosomes, lysosome-related organelles that can be divided into four stages depending on their degree of maturation [2]. Stage I pre-melanosomes lack pigment but develop distinct fibrillar structures in a Pmel17-dependent process during stage II. Tyrosinase and other enzymes of melanogenesis reach stage II melanosomes via endosomal intermediates and initiate the production of melanin; these melanosomes are deposited on the fibers, resulting in their thickening and blackening with maturation to stage III. Melanin synthesis and deposition continue until all of the internal structure is masked in stage IV [3,4]. Modulators of melanin production include proteins involved in melanosome structure (i.e., Pmel17, MART-1), proteins involved in melanin synthesis and melanosome pH regulation (i.e., tyrosinase, TYRP1, TPC2), and proteins required for melanosome transport and distribution (e.g., Rab27a, myosin Va, melanophilin) [3,4]. Melanosomal pH is regulated by the vacuolar proton ATPase, Na^+^/H^+^ exchangers, SLC24A5 (Na^+^/K^+^/Ca^2+^ exchanger 5), and two-pore channel 2 (TPC2) [4,5,6,7,8]. TPC2, a cation channel permeable for sodium and calcium, expressed in late endosomes (LE), lysosomes and melanosomes, regulates pigmentation through two fundamental determinants of melanosome function: pH and size [7]. Different steps of melanogenesis are regulated by pH. First, the activity of tyrosinase, the rate-limiting enzyme for the production of melanin the optimal pH of which is 6.8 [9] with greatly reduced activities at a more acidic pH [5], while differences between monophenol oxidase and diphenol oxidase activities of tyrosinase in their pH dependence should be noted. Second, the activation of metatyrosinase (reduction of Cu II), the initial step of melanogenesis, is also highly pH-dependent. Third, the non-enzymatic DOPA-producing reaction, the redox exchange, is also strongly dependent on pH [10,11,12].

Two human TPC2 gain-of-function (GOF) variations were identified as associated with blond hair color: rs35264875 (encoding M484L), which results in an increased sensitivity to the endogenous TPC2 ligand PI(3,5)P_2_, while rs3829241 (encoding G734E) results in reduced channel inhibition by ATP [8,10,13]. Both variations are mainly found in Europeans, particularly in blond-haired Northern Europeans [14]. On the contrary, knockout of TPC2 results in a strong increase in melanin production in both primary human melanocytes and in pigmented melanoma cells (i.e., MNT-1) [7].

It is well-established that melanin is one of the major protective factors against UV radiation mediated DNA damage that results in melanoma development [15]. Besides, individuals with a higher ratio of pheomelanin to eumelanin in their skin and hair, that is, blond- and red-haired individuals, have a greater risk for melanoma than black- or brown-haired individuals (by a factor of 2–4) [16,17].

In addition to melanosomes, TPC2 is also expressed in LEs/lysosomes, while expression of its relative TPC1 appears to dominate in endosomes. A role of TPCs in cancer cell migration, invasion and proliferation has been convincingly established in the last couple of years, attributed to its function in endolysosomes [18,19,20,21,22,23,24,25,26]. While common consensus suggests that knockout, knockdown, or pharmacological inhibition of TPCs, and in particular TPC2, reduces cancer cell migration, invasion and proliferation, including melanoma cells, some report otherwise.

## 2. Role of MITF in Melanoma and Pathways Implicated

### 2.1. MITF

Microphthalmia-associated transcription factor (MITF) is a central player of melanocyte survival, function and development [27,28,29]. It belongs to the MiT/TFE family of transcription factors in vertebrates, consisting of four distinct but closely related and evolutionary conserved members, including MITF, transcription factor EB (TFEB), TFE3 and TFEC. Structurally, MITF encodes a basic–helix–loop–helix leucine zipper (bHLH-ZIP) transcription factor, thereby exerting its function by regulating genes involved in cell cycle progression and differentiation, a role sustained throughout the process of melanogenesis and in melanoma [30,31,32]. Mutations in MITF are associated with Tietz albinism-deafness syndrome and Waardenburg syndrome type 2A [33,34], and amplification of MITF is found in 15–20% of human metastatic melanomas and has been linked to poor survival. The M-MITF isoform is the predominant isoform in 80% of human melanomas [28,29,35].

### 2.2. Rheostat Model of MITF

**Figure 1:**
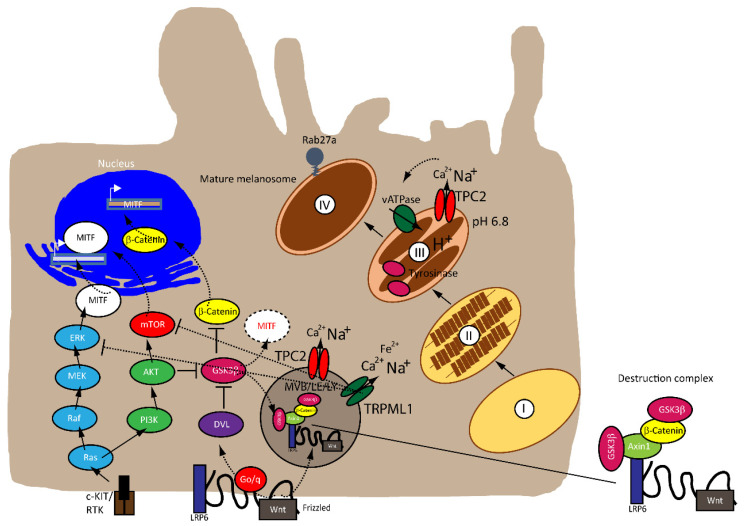
Signaling pathways in melanoma cells involved in MITF regulation and subcellular localization of TRPML1 and TPC2. Several pathways regulate MITF expression: RAS/RAF/MEK/ERK, PI3K/AKT and Wnt/GSK3β/β-Catenin. GSK3β is a negative regulator of MITF expression and promotes proteasomal degradation of MITF. GSK3β degradation in endolysosomes is enhanced by Wnt signaling. Tyrosinase activity depends on melanosomal pH and is regulated by TPC2 activity in melanosomes. Loss-of-function of TPC2 in endolysosomes and melanosomes results in increased GSK3β and decreased MITF protein levels as well as increased tyrosinase activity and melanin content.

MITF is regulated by numerous factors that exercise tight control on both its transcriptional and post-translational levels (i.e., ubiquitination, acetylation and sumoylation), given that it is downstream of several pathways [36,37], as illustrated in Figure 1. A rheostat model of MITF in melanoma has been proposed by Carreira et al. (2006), where MITF yields three phenotypes, hence varied cellular responses in melanoma based on its activity. At the highest state where c-AMP induction leads to peak MITF activity, melanoma cells express differentiation target genes (i.e., Tyrosinase and MART1), giving rise to a pigmented phenotype and undergoing terminal differentiation. In contrast, the cells at the intermediate activity level are at a reversible proliferative state where enough MITF is expressed to activate MITF genes linked to survival (i.e., BCL2 and CDK2), concomitantly suppressing p27Kip1 expression through the regulation of Dia1, preventing differentiation. While MITF at its lowest activity brings about highly invasive cells exhibiting stem cell-like properties and low proliferative and pigmentation capacities that undergo p27Kip1-mediated G1 arrest [38,39]. Nonetheless, the rheostat model is controversial for many reasons. First, MITF is a downstream target of numerous signaling pathways and is subject to diverse post-translational modifications, which can direct MITF to different sets of target genes that regulate different functions in melanoma, dependent on physiological context [31,33]. Furthermore, findings that proliferative and invasive phenotypes are mutually exclusive has been disputed by Haass et al. (2014) [40]. Wellbrock et al. (2015) describe how the comparable expression of MITF can carry out opposing functions depending on the setting [38,41]. Finally, Carreira et al. (2006) themselves specify that the roles assigned to “MITF activity” are in fact limited to expression levels using overexpression and depletion models, and most data have been established using in vitro models [38]. For the purpose of this review, we will be discussing two prominent pathways involved in the regulation of MITF: MAPK and Wnt.

### 2.3. MITF and MAPK

The mitogen-activated protein kinase (MAPK) pathway is dysregulated in 90% of melanoma with 50% of patients harboring activating mutations in B-RAF, most commonly the BRAF^V600E^ mutation—an amino acid substitution from valine (V) to glutamic acid (E)—while 28% of patients carry mutations in N-RAS, leading to a constitutively hyperactivated MAPK signaling and increased melanoma cell survival, proliferation, migration, invasion, metastasis and angiogenesis. Consequently, the targeted therapies that are currently available have focused on developing specific small molecule tyrosine kinase inhibitors (TKI) for BRAF (BRAFi) and MEK (MEKi), which have shown improved survival rates [42,43,44,45,46].

The relationship between MITF and the MAPK signaling hub is largely relevant through p-ERK phosphorylation and the mutational status of B-RAF. Garraway et al. (2006) have observed that MITF gene amplification in metastatic melanoma coincides with BRAF^V600E^ mutation and p16 inactivation, promoting tumor growth and survival, while MITF reduction exposes melanoma to conventional chemotherapeutic sensitization [29]. Van Allen et al. (2014) have shown that patients relapsed on BRAF/MEK inhibitor therapy exhibit increased gene dosage of MITF [47]. These results have been reproduced in vivo by Lister et al. (2014), where reduced expression levels of MITF in BRAF^V600E^ mutated melanoma in a zebrafish model have led to tumor regression [48]. To date, two closely linked ERK-mediated phosphorylation sites on MITF have been found: S73 and S409, which target MITF for proteasomal degradation, shortening its half-life [49,50]. While single mutations on these sites, S73A and S409A, have been found to reduce the transcriptional activity of MITF, the S73A/S409A double mutation, although stable, demonstrated a complete inhibition of the transcription and transactivation of MITF [50,51]. In contrast to the S73 phosphorylation site of MITF, which could exclusively serve as an MAPK pathway target, phosphorylation events in downstream pathways, other than the MAPK, such as protein kinase A (PKA) and GSK3 take place at S409, in turn being responsible for MITF activation/degradation and serving as a focal point for the divergent pathways controlling it [50,52].

### 2.4. MITF and Canonical Wnt Pathway

The Wnt signaling pathway is divided into canonical β-catenin dependent and non-canonical β-catenin independent branches and is involved in regulating cellular homeostasis and development. Mutations of components of this pathway contribute to a number of pathologies, including familial exudative vitreoretinopathy, tooth development defects, Robinow syndrome, bone density defects, Alzheimer’s disease, and different types of cancers, such as melanoma, hepatocellular carcinoma (HCC), ovarian cancer, breast cancer and prostate cancer [53,54,55,56]. In melanoma, aberrations of the canonical Wnt pathway are directly linked to MITF and the rheostat model described above. The key player is β-catenin, which regulates genes of the melanocyte lineages and facilitates early-stage melanocyte transformation by blocking cellular senescence and increased proliferation. In melanoma cells, β-catenin activates proteins involved in melanogenesis and pigmentation such Melan-A, dopachrome tautomerase (DCT) and tyrosinase, all through MITF [55,57,58,59].

Recently, Ploper et al. (2015) have established a clear link between the endolysosomal machinery, MITF and the canonical Wnt signaling pathway in melanoma. Analyzing RNA microarray databases followed by gene set enrichment analysis of numerous melanoma cell lines, the authors found a positive correlation between expression levels of MITF and lysosomal genes, independent of TFEB levels, claiming that MITF, via direct activation of the CLEAR element in lysosomal genes, induces their transcription. Moreover, MITF expression expanded multivesicular bodies (MVBs) and LE, without affecting the number of lysosomes in the C32 melanoma line, enhancing Wnt signaling and the proliferation of these cells. While the S409 phosphorylation site has been described above, Ploper et al. (2015) have discovered three novel phosphorylation sites on the C-terminus of MITF: S397, S401 and S405, which promotes the proteasomal degradation of MITF by GSK3β. The authors have proposed the following mechanism of action: during the inactive state of the Wnt pathway, GSK3β would phosphorylate MITF on the sites uncovered above, rendering MITF unstable for undergoing proteasomal degradation. Upon activation of the pathway, GSK3β would be inhibited, stabilizing MITF, which in turn would induce the translocation of the destruction complex components (i.e., Axin1, p-β-catenin, GSK3β) to CD63^+^ MVBs and LE. MITF would then accumulate in the nucleus, activating the lysosomal genes and contributing to endolysosomal biogenesis. In turn, this generates a positive feedback loop by inducing and increasing the number of MVBs and LE that sequester the destruction complex further, without undergoing proteolysis [52].

## 3. Endolysosomal Cation Channels in Melanoma

**Figure 2:**
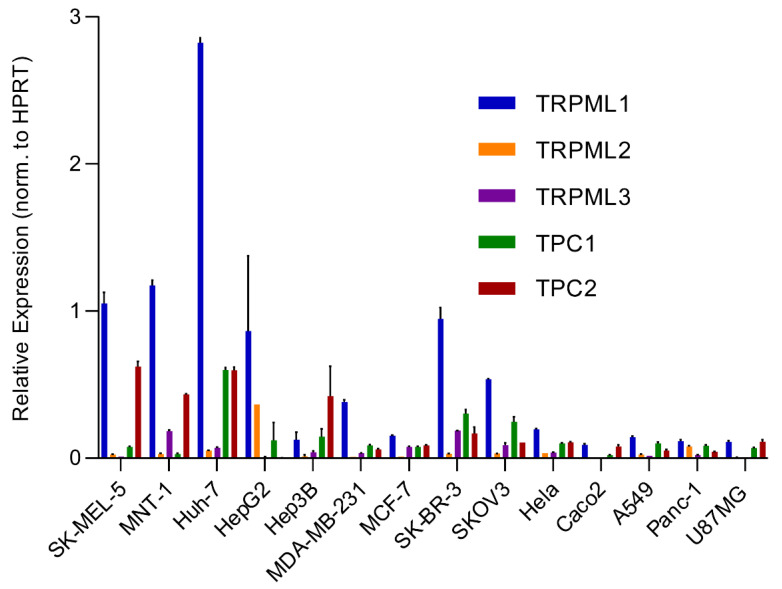
Gene expression profile of endolysosomal cation channels in different cancer cell lines. The expression levels of endolysosomal cation channels TRPML1, TRPML2, TRPML3, TPC1 and TPC2 were assessed in different cancer cell lines using real-time quantitative Reverse Transcription PCR (qRT-PCR): melanoma (SK-MEL-5 and MNT-1); hepatocellular carcinoma (HUH-7, HebG2, and Hep3B); breast cancer (MDA-MB-231, MCF-7 and SK-BR-3); colon adenocarcinoma (Caco-2); ovarian cancer (SKOV-3), cervical adenocarcinoma (Hela); pancreatic ductal adenocarcinoma (Panc-1); glioblastoma (U87MG); lung adenocarcinoma (A-549). Melanoma and HCC lines consistently showed the highest expression levels of TRPML1 and TPC2. Primer sequences and qRT-PCR were performed as discussed previously [60].

An analysis of the expression of different endolysosomal cation channels in a number of cancer cell lines using real-time quantitative reverse transcription PCR (qRT-PCR), including melanoma, hepatocellular carcinoma, breast cancer, colon adenocarcinoma, ovarian cancer, cervical adenocarcinoma, pancreatic ductal adenocarcinoma, glioblastoma and lung adenocarcinoma, is shown in Figure 2. The cell lines expressing particularly high levels of TPC2 are HCC and melanoma lines. Likewise, very high levels are found in melanoma lines for TRPML1. In the following, we will focus on these two channels.

### 3.1. TPC2

Two recent works have examined the role of TPC2 in melanoma cells in more detail [24,26]. D’Amore et al. (2020) used human CHL-1 amelanotic melanoma cells (derived from a metastatic site: pleural effusion) while Netcharoensirisuk et al. (2021) used the highly pigmented human MNT-1 melanoma cell line (likewise derived from a metastatic site: lymph node). The latter study used TPC2 knockout MNT-1 melanoma cells previously published by Ambrosio et al. (2016), confirming that TPC2 knockout results in a strong increase in melanin content. It was further shown that knockout of TPC2 in MNT-1 cells results in a significant decrease in cell proliferation, migration and invasion [7,19,25]. In addition, an increase in the activity and expression of tyrosinase was found while transcript levels were unchanged. Expression levels of other proteins involved in melanogenesis or regulated by MITF, such as dopachrome tautomerase (Dct, TYRP2), Ras-related protein Rab-27A (Rab27a) or premelanosome protein (PMEL), were normal. This exclusive increase in tyrosinase activity and expression is surprising but even more surprising is the finding that protein levels of MITF were decreased in TPC2^−/−^ versus WT cells.

Besides the involvement of MAPK and Wnt/GSK3β/β-Catenin pathways described above, melanin formation is also triggered by melanocyte-stimulating hormone (MSH), a peptide hormone encoded by the proopiomelancortin gene (POMC). MSH binding to MC1R results in the induction of MITF via the cAMP response element-binding protein (CREB). In the TPC2^−/−^ MNT-1 cells, the expression levels of ERK, AKT, and CREB were found to be unchanged while the expression of GSK3β was increased [20]. It was thus concluded that the increased level of GSK3β results in reduced degradation of the GSK3β containing destruction complexes in endolysosomes, leading to increased GSK3β-dependent MITF degradation. These findings were recapitulated in MNT-1 cells treated with blockers for TPC2 [26] and it was found that flavonoid blockers of TPC2, such as naringenin [61], pratensein or duartin [26] like genetic loss of TPC2, increases melanin content and decreases proliferation, migration and invasion in a TPC2 dependent manner. In TPC2^−/−^ cells, no significant effects of the compounds were seen. In sum, Netcharoensirisuk et al. (2021) concluded that melanoma cell proliferation, migration and invasion are inversely correlated with TPC2-dependent melanin production in MNT-1 cells as the reduction of TPC2 expression increases melanin content but decreases proliferation, migration and invasion. It was suggested that this is the consequence of independent mechanisms: the regulation of MITF protein levels through interference with the endolysosomal activity of TPC2 and endolysosomal GSK3β degradation on the one hand and, on the other hand, the regulation of tyrosinase activity in melanosomes, independent of MITF by indirect interference with the melanosomal proton pump activity (decreased driving force for the pump due to absent or reduced TPC2 activity, resulting in reduced proton uptake by melanosomes, leading to less acidic melanosomal pH, increased tyrosinase activity, and eventually increased melanin production).

Clearly, this dual activity of TPC2 in melanosomes and endolysosomes is a special feature for TPC2 in melanoma cells, which requires further attention to understand its full potential as a possible drug target to treat melanoma.

In the work by d’Amore et al. (2020), amelanotic cells were used. The dual expression of TPC2 in melanosomes and endolysosomes was not discussed. The authors generated a CHL-1 TPC2 knockout line and found that the knockout cells were more invasive and the expression of MITF was increased in the TPC2^−/−^ KO cells as compared to the WT, in contrast to the study by Netcharoensirisuk et al. (2021). A link was shown to the Hippo signaling pathway, which regulates several biological processes including cellular proliferation and survival, and differentiation was postulated. Dysregulation of this pathway, resulting in an increase in YAP/TAZ activity, is associated with cancer, promoting, for example, hyper-proliferation, cellular invasion and metastasis. While YAP/TAZ expression was unchanged in the TPC2 knockout line, some YAP/TAZ target genes were found to be increased, including ANKRD1, CYR61 and CTGF. The drastic differences between these two studies may be due to the amelanotic versus highly pigmented nature of the used melanoma lines, the status of the B-RAF mutation, MITF or other upstream pathways controlling MITF [24,26].

### 3.2. TRPML1

TPCs are functionally related to another group of non-selective, endolysosomal cation channels—the TRPMLs or mucolipins—with three members in the mammalian genome: TRPML1, 2 and 3. Roles in cancer for all three channels have been proposed, excellently summarized in a number of recent reviews [20,62,63,64].

Kasitinon and colleagues (2019) have recently screened ion channels and transporters throughout the genome to identify those required by human melanoma cells but not by normal melanocytes, and found that TRPML1 deficient melanoma cells exhibit decreased proliferation, tumor growth and survival [65]. The growth of healthy human melanocytes was unaffected by the loss of TRPML1. They further found TRPML1 to be required in melanoma cells to negatively regulate the MAPK pathway and mTORC1 signaling. mTORC1 promotes cellular proliferation by activating anabolic pathways and by inactivating catabolic pathways such as autophagy, and the MAPK pathway regulates MITF, introduced above as a major regulator of melanoma proliferation and progression. While Kasitinon et al. (2019) found that the deletion of TRPML1 in melanoma increases p-ERK and mTORC1 signaling, they did not discuss their findings in the context of MITF. TRPML1 has been postulated before to promote mTORC1 activity [66,67,68]. According to the concept by Kasitinon et al. (2019) of the augmentation of MEK-ERK signaling in TRPML1 deficient cells, knockout of TRPML1 would promote MITF transcription and expression [65].

## 4. Summary and Conclusions

The studies discussed here lack information about the role of the endolysosomal cation channels in melanoma development. More melanoma cell lines should be tested in the future as contradictory findings in MNT-1 (highly pigmented) versus CHL-1 (amelanotic) cell lines need further attention. In particular, a clear mechanistic overview of the different signaling pathways involved upstream of MITF—the key player in melanoma—would be of utmost importance.
